# Clinical results and toxicity for short-course preoperative radiotherapy and total mesorectal excision in rectal cancer patients

**DOI:** 10.1093/jrr/rru089

**Published:** 2014-10-23

**Authors:** Florian Sterzing, Frieder Hoehle, Alexis Ulrich, Alexandra Jensen, Jürgen Debus, Marc Muenter

**Affiliations:** 1 Department of Radiation Oncology, University Hospital Heidelberg, INF 400, 69120 Heidelberg, Germany; 2 Clinical Cooperation Unit Radiation Oncology, German Cancer Research Center (DKFZ), Heidelberg, Germany; 3 Department of Radiation Oncology, University Hospital Marburg, Germany; 4 Department of Surgery, University Hospital Heidelberg, Germany; 5 Department of Radiation Oncology, Katharinen Hospital, Stuttgart, Germany

**Keywords:** rectal cancer, short-course radiotherapy, radiation toxicity; local control; sexual dysfunction

## Abstract

Short-course preoperative radiotherapy (SCPRT) is an alternative method to chemoirradiation for patients with Stage II and III rectal cancer when no downsizing is needed, but there is still widespread reluctance to use this method because of fear of side effects from high-fraction doses. This paper reports on a single institution patient cohort of operated rectal cancer patients after SCPRT, evaluated for chronic adverse effects, local control, progression-free survival and overall survival. Altogether, 257 patients were treated with SCPRT and surgery including total mesorectal excision (92% total mesorectal excision = TME) between 2002 and 2009. Local control and survival were analyzed. Chronic adverse effects for 154 patients without local relapse were evaluated according to the NCI–CTCAE version 4.0 classification, with a median follow-up of 48 months. We found a 5-year disease-free survival (DFS) and overall survival (OS) of 71%. The 5-year estimated local control (LC) rate was 94%. A positive resection margin was found in 4% of the patients and was significantly correlated with decreased DFS, OS and LC. Chronic adverse effects were reported by 58% of the patients, of which 10% were Grade 3 toxicities. The most frequent Grade 2 toxicity was stool incontinence (13%). Sexual dysfunction was found in 36% of the patients (31% Grade 1 or 2, and only 5% Grade 3). SCPRT combined with TME produced excellent LC rates together with a low rate of high-grade chronic adverse effects.

## INTRODUCTION

Total mesorectal excision (TME) has become the standard operative procedure for rectal cancer of the middle and lower third of the bowel [1]. Through improvement in the surgical standard, local relapse rates of 5–12% in Stage II/III patients have been achieved, and in selected series even below 3% [2].

Long-course chemoirradiation and short-course radiotherapy are two regimens that have proven to be effective in the neoadjuvant setting by reducing the risk of local relapse by ∼50% [3]. The benefit for overall survival (OS), however, has vanished in most trials using TME [4]. Only the update of the Dutch TME trial showed a statistically significant difference in OS for Stage III patients after 10 years (50% vs 40% in favor of the irradiation group) [5].

Recent trials have strengthened the evidence supporting the use of short-course radiotherapy [6]. Nevertheless, there is still considerable reluctance to use this regimen in some parts of the world as a result of uncertainty about the potential side effects of higher single doses. The early SCPRT trials did indeed find a higher complication rate after irradiation [7]. However, this was using a 30-year-old radiotherapy technique from today's perspective, with the APPA technique and the upper field border at L2. This differs greatly from today's 3D planned multifield technique with the upper border at the promontorium [8].

The Stockholm II trial showed no increased toxicity rate in the radiotherapy group due to the introduction of three- or four-field box techniques [9]. Furthermore, the reported toxicity is lower than in classically fractionated chemoirradiation, and patient compliance could be increased [10–13]. The current guidelines allow both modalities, depending on the clinical goal. This retrospective analysis describes the outcome and toxicity experienced by rectal cancer patients treated with SCPRT in Heidelberg between 2002 and 2009.

## METHODS

### Patient inclusion criteria

Patients who were treated with 5 × 5-Gy preoperative radiotherapy with curative intention for histologically confirmed rectal cancer (defined as a tumor within 16 cm of the anal verge) in the University Hospital of Heidelberg between 2002 and 2009 were included in this study. After approval by the ethics committee, data for the 257 patients were retrospectively reviewed. All living patients were contacted for completing a questionnaire about relevant long-term gastrointestinal, urological, sexual and dermatological toxicities graded according to NCI–CTCAE version 4.0.

Of the 257 patients, 237 had a postoperative pathological cancer Stage I–III; in addition, a subset of 20 patients with a Stage IV cancer was analyzed separately. Table 1 shows the details of the patients. The patients in Stage I–III had a median age of 67 years (38–87); 93 were female and 144 were male. Exact distance from the anal verge was available in 232 of the 237 patients. Of these, 23 patients had a tumor in the upper third (12–16 cm), 144 in the middle third (6–12 cm) and 65 in the lower third ( <6 cm).

**Table 1.** Patient and treatment characteristics

Total number	257			
Postoperative stage	I	II	III	IV
	54 (21%)	69 (26.8%)	114 (44.4%)	20 (7.8%)
Distance from anl verge	< 6 cm	6–12 cm	12–16 cm	unknown
	69 (26.9%)	158 (61.5%)	25 (9.7%)	5 (1.9%)
Age	median 67 years (min 38, max 87)			
Sex	female	male		
	96 (37.4%)	161 (62.6%)		
Interval to operation	median 7 days after first fraction (min 6, max 39) in 93% of patients within 10 days after 1st fx			
Surgical procedure	LAR	APR	other	
	227 (88.3%)	21 (8.2%)	9 (3.5%)	
	TME			
	237 (92.2%)			
Resection margin	R0	R1	R2	unknown
	237 (92.2%)	12 (4.7%)	0	8 (3.1%)
Adjuvant chemotherapy	Stage II	yes	no	
		9/69	60/69	
		FOLFOX	Capecitabine	5FU/leucovorin
		44%	44%	11%
	Stage III	yes	no	
		55/114	59/114	
		FOLFOX	Capecitabine	5FU/leucovorin
		27%	24%	49%
	Stage IV	yes	no	
		16/20	4/20	
		FOLFOX	FOLFOX + antibody	
		62.5%	37.5%	

### Patient exclusion criteria

Patient exclusion criteria were: concurrent predispositions or diseases such as familiar adenomatous polyposis (FAP), hereditary non-polyposis colon cancer (HNPCC), Crohn's disease and ulcerative colitis, in order to exclude these diseases as confounding factors; disease relapse, to rule out effects of further therapies; and radiotherapy within a palliative setting.

### Radiotherapy

All 257 patients received a neoadjuvant radiotherapy of 25 Gy in five fractions applied in consecutive working days. All patients received computed tomography-based 3D conformal radiotherapy with a multileaf collimator (MLC) and were immobilized in a belly board in prone position. The superior border of the clinical target volume (CTV) was the promontorium, the inferior border was 3 cm below the distal tumor end, and the lateral border was the pelvic wall. The anterior field border was at least 2 cm from the tumor and the posterior border was the posterior surface of the sacral bone. The CTV included the rectum, mesorectum, common and internal iliac vessels with a 1-cm margin, presacral space, posterior part of the internal obturator muscle and 1–2 cm of the bladder. The clinical to planning target volume (PTV) margin was 5 mm. Treatment was delivered with linear accelerators with photon beams of 6–18 MV. A 3-field box with two lateral beams and one posterior beam was used in most cases.

### Surgery

Surgery was performed after a median interval of 7 days after the beginning of radiotherapy (range 6–39 days). In 93% of the patients, surgery was undertaken within 10 days after the start of radiotherapy. Most of the operations (91%) were performed in the University Hospital of Heidelberg; 92% of the surgical procedures were total mesorectal excisions (TMEs), 2% partial mesorectal excisions, and in 6% no exact procedure could be identified. A low anterior resection was performed for 87% of the patients, an abdominoperineal extirpation for 8%, a high anterior resection for 2%, a Hartmann resection for 2%, and a complete proctocolectomy for 1%. R1 resection status was found in 4.7% of the patients.

### Adjuvant chemotherapy

Of the 108 Stage III patients, 55 received adjuvant chemotherapy: 5FU and leucovorin was used in 49%, FOLFOX (leucovorin calcium (folinic acid), fluorouracil and oxaliplatin) in 27% and capecitabine in 24% of the patients.

### Follow-up and statistics

Toxicity assessment was performed during routine follow-up examinations, which were carried out every 3 months in the first 2 years and every 6 months in the following years. The questionnaires were completed at varying time-points after treatment, ranging from 12 months to 88 months. For those patients who were initially scored according to CTC version 3.0, results were adapted for the current version (4.0).

Disease-free survival (DFS) is the percentage of people in the trial who are alive and cancer free after a specified number of years. OS is the number of people alive, with or without signs of cancer.

## RESULTS

### Survival and local control

**Fig. 1.**
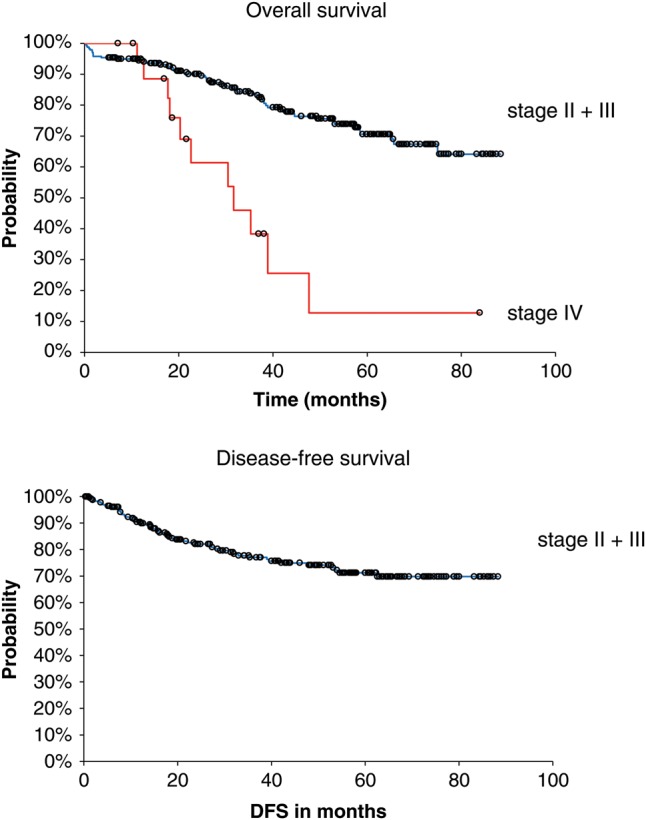
Kaplan–Meier estimation of overall survival and disease-free survival.

**Fig. 2.**
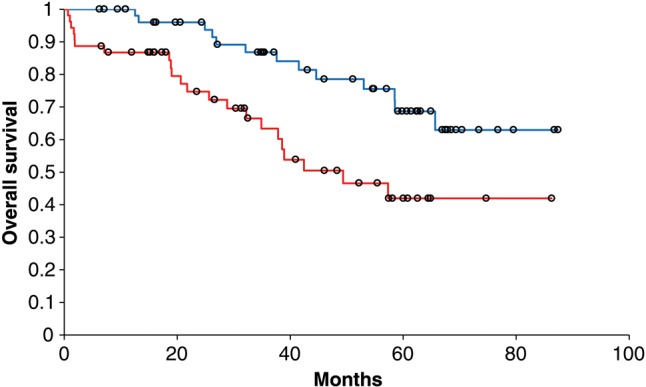
Overall survival for Stage II or III cancer patients receiving adjuvant chemotherapy (blue) or no adjuvant chemotherapy (red).

The median follow-up was 48 months (5.2–88.3 months). The Kaplan–Meier estimation of 5-year OS for Stage I–III patients was 71% (95% confidence interval 63–77%); the curves are shown in Fig. 1. The estimated 5-year OS for Stage IV patients was 13% (1–41%). No local relapse was found in Stage I patients. The estimated LC in Stage II patients was 94% after 5 years, and for Stage III patients 91%. For patients with positive resection margins (*n* = 10), 5-year LC was only 60%. Stage-dependent patterns of disease relapse are displayed in Table 2. The 3-year distant metastases-free survival was 80%. Figure 2 displays the OS of Stage III patients who received adjuvant chemotherapy vs no adjuvant chemotherapy. OS was significantly different, with a 2-year OS, 3-year OS and 5-year OS of 96%, 87% and 69% in the chemotherapy group and 75%, 63% and 42% in the non-chemotherapy group (*P* = 0.0038). No statistically significant difference for LC or distant metastases-free survival could be found between the two groups.

**Table 2.** Patterns of disease relapse after 5 years according to tumor stage

	No relapse	Relapse	Local relapse	Distant metastases	Local and distant
UICC I (*n* = 54)	53	1	0	1	0
	98.2%	1.9%	0%	1.9%	0%
UICC II (*n* = 69)	60	9	2	6	1
	87.0%	13.0%	2.9%	8.7%	1.5%
UICC III (*n* = 113)	67	46	5	39	1
	59.3%	40.7%	4.4%	34,0%	0.9%
Total (*n* = 236)	180	56	7	46	2
	76.3%	23.7%	3.0%	19.5%	0.8%

### Perioperative morbidity and mortality

Of the total number of 257 patients, 11 died within the three months following surgery (4.3%), and the 30 days perioperative mortality was 1.9%. Six died due to septic complications, two passed away due to cardiorespiratory failure, and one patient died due to massive mucositis associated with adjuvant chemotherapy. Two patients had an unclear cause of death.

Interventions for anastomic insufficiency were needed by 15 patients (6.5%); two patients developed an ileus, two patients a fistula and six patients severe infections. The total perioperative Grade 3 and 4 toxicity was 10.5%.

### Chronic adverse effects

The following adverse events were observed in the cohort of patients that were locally controlled. Of the 164 relapse-free patients (94%), 154 completed the questionnaire (95 patients were male and 59 female), and 26 patients had a stoma at the time of the interview.

In total, 90 patients (58%) reported long-term side effects. Grade 3 effects were found in 10%, Grade 4 or 5 toxicity was not found. Details of the toxicities are displayed in Table 3. Gastrointestinal problems accounted for 64% of the chronic adverse effects, and 91% of these were Grade 1 and 2. Only five patients experienced Grade 3 toxicity: two patients with diarrhea, two with a fistula and one with necrosis of the neorectum. Grade 2 fecal incontinence was reported by 13% of the patients. Only one Grade 3 genitourinary toxicity was reported, which was a urethral stricture. Urinary incontinence Grade 1 and 2 was reported in 6% of the patients. Bone fractures occurred in three patients; these were two femoral neck fractures and one fracture of the fifth lumbar vertebra.

**Table 3.** Long-term toxicity after SCPRT in 154 patients

Gi	No adverse effects	Adverse effects total	Grade 1	Grade 2	Grade 3
Proctitis	138 (95%)	7 (5%)	2 (1%)	5 (3%)	0 (0%)
Stool incontinence	89 (69%)	40 (31%)	22 (17%)	17 (13%)	1 (1%)
Rectal fistula	141 (97%)	4 (3%)	2 (1%)	0 (0%)	2 (1%)
Rectal bleeding	143 (99%)	2 (1%)	2 (1%)	0 (0%)	0 (0%)
Rectal necrosis	144 (99%)	1 (1%)	0 (0%)	0 (0%)	1 (1%)
Rectal pain	137 (94%)	8 (6%)	4 (3%)	4 (3%)	0 (0%)
Rectal stenosis	140 (97%)	5 (3%)	0 (0%)	5 (3%)	0 (0%)
Anal pain	140 (97%)	5 (3%)	2 (1%)	3 (2%)	0 (0%)
Anal dermatitis	138 (95%)	7 (5%)	5 (3%)	2 (1%)	0 (0%)
Flatulence	127 (98%)	2 (2%)	1 (1%)	1 (1%)	–
Diarrhea	122 (95%)	7 (5%)	2 (2%)	3 (2%)	2 (2%)
Stomal ulceration	24 (92%)	2 (8%)	0 (0%)	2 (8%)	0 (0%)
**Gu**	**No adverse effects**	**Adverse effects total**	**Grade 1**	**Grade 2**	**Grade 3**
Cystitis	153 (99%)	1 (1%)	0 (0%)	1 (1%)	0 (0%)
Pollakisuria	152 (99%)	2 (1%)	1 (1%)	1 (1%)	–
Urinary incontinence	145 (94%)	9 (6%)	4 (3%)	4 (3%)	1 (1%)
Urinary retention	150 (97%)	4 (3%)	2 (1%)	2 (1%)	0 (0%)
Strangury	150 (97%)	4 (3%)	3 (2%)	1 (1%)	–
Obstruction	152 (99%)	2 (1%)	0 (0%)	2 (1%)	0 (0%)
Bladder spasm	153 (99%)	1 (1%)	0 (0%)	1 (1%)	0 (0%)
**Sexual**	**No adverse effects**	**Adverse effects total**	**Grade 1**	**Grade 2**	**Grade 3**
Patients (122)	78	44	10	28	6
%	64%	36%	8%	23%	5%
Male 81	45	36	9	26	1
%	56%	44%	11%	32%	1%
Female 41	33	8	1	2	5
%	80%	20%	2%	5%	12%
	**No adverse effects**	**Adverse effects total**	**Grade 1**	**Grade 2**	**Grade 3**
Female loss of libido	37 (90%)	4 (10%)	2 (5%)	2 (5%)	–
Male loss of libido	62 (77%)	19 (23%)	13 (16%)	6 (7%)	–
Erectile dysfunction	52 (64%)	29 (36%)	6 (7%)	22 (27%)	1 (1%)
Ejaculation problems	75 (93%)	6 (7%)	2 (2%)	4 (5%)	–
Menorrhea problems	38 (93%)	3 (7%)	0 (0%)	0 (0%)	3 (7%)
Dyspareunia	36 (88%)	5 (12%)	2 (5%)	1 (2%)	2 (5%)

Flatulence, diarrhea and stool incontinence data only for patients without a stoma (*n* = 128). Graded according to CTCAE version 4.0.

Of the 154 patients, 122 could be evaluated with regard to sexual dysfunction. The rest did not wish to answer questions on this topic. Of these, 44 patients (36%) reported sexual dysfunctions; 38 of these were Grade 1 and 2—44% of the male patients reported problems, while only 20% of the female patients reported problems. All sexual dysfunctions were reported in women younger than 70 years, and for this specific subgroup we calculated a 35% chance of sexual dysfunction. Only two female patients reported Grade 3 dysfunction with painful dyspareunia. Erectile dysfunction (ED) was found in 37% of the men, 27% being Grade 2 and 1% Grade 3. Overall, 35% of the patients reported no more sexual activity, 31% reduced activity since onset of disease and therapy, 19% found no change and 15% did not want to answer. No differences for sexual or gastrointestinal long-term toxicities were found between the chemotherapy and the non-chemotherapy group.

## DISCUSSION

This is one of the largest reported single-center series of patients treated with SCPRT. Our LC and survival rates are comparable with the results of large randomized trials such as the MRC CR07 trial, which compared SCPRT with selective postoperative chemoirradiation [4]. The rate occurrence of distant metastases of 20.4% and the LC rate of 94% compare favorably with the results of the Dutch TME trial and other prospective trials [14–17].

Of our 237 patients, 54 were diagnosed with Stage I rectal cancer in pathological examination after preoperative Stage II or III. This 23% rate of overstaging is comparable with the Sauer trial (with a rate of 25%), although a considerable fraction of this was a result of partial and complete remissions following neoadjuvant chemoirradiation [18]. Transrectal endoscopic ultrasound is reported to be correct in nodal staging 62–87% and T-stage 80–95% [19]. This is in part caused by desmoplastic reactions resulting from local inflammatory reactions that can compromise T2–3 discrimination [20].

The rate of positive resection margins was low in our series (10 patients). The low estimated OS rate of 19% after 5 years reflects both the advanced tumor stage and the importance of clear resection margins. This unfavorable outcome was the result of preoperative understaging and clearly shows the limits of SCPRT in comparison with chemoirradiation.

The described outcome differences in this retrospective study for adjuvant chemotherapy have to be interpreted with caution. The group of Stage III patients that did not receive adjuvant chemotherapy contains all the individuals that had experienced severe perioperative morbidity or died perioperatively. In addition, this group had older patients with severe comorbidities. In other words, the better results for the chemotherapy group surely are influenced by the fact that we are looking at younger and fitter patients without complications. Yet a difference in distant metastases-free survival could not be found between the two groups.

The use of perioperative radiotherapy is under discussion, since standardized TME has changed the risk of local relapse. While perioperative radiotherapy still improves LC, it comes at the cost of toxicity that cannot be neglected [5, 21].

With regard to the toxicity of SCPRT, we found very low rates of high-grade side effects. The rate of Grade 3 or 4 toxicity experienced within the first 3 months following SCPRT was 10.5% in our patient cohort. Chronic fecal incontinence was found in 17%, 13% and 1% of patients with Grade 1, 2 and 3 toxicity, respectively. Because of the range of classifications and time intervals in different publications, a structured comparison among trials is influenced by various confounding factors. In the Dutch TME trial, 62% of the irradiated patients showed fecal incontinence, as opposed to 38% of the non-irradiated group. Pad wearing as a result of incontinence was reported in 56% vs 33% of patients, and satisfaction with bowel function was worse in the radiotherapy group [22]. The MRC CR 07 trial revealed a percentage of unintended stool loss after 2 years of 53% in the SCPRT group vs 37% in the selective postoperative group (*P* = 0.004) [23]. Ulrich *et al*. found an overall fecal incontinence rate of 42.1% in a cohort of more than 600 patients, both non-irradiated and irradiated. The risk for stool incontinence was associated with SCPRT; however, confounding factors such as tumor size and distance from the anal verge make this correlation difficult to judge [24]. Bujko *et al.* reported a 72% rate of anorectal dysfunction after SCPRT, 8% of which was high grade. In total, there was no significant difference between short- and long-course radiotherapy [12]. Sphincter preservation in our trial was achieved in two-thirds of lower-third rectal cancer, but rates of fecal incontinence were higher than in other localizations (total: 40% vs 31%, Grade 2: 28% vs 13%), probably caused by a full dose of irradiation to the sphincter in combination with deep resection.

The reported sexual dysfunction rate in the literature has a wide range: 23–69% in men and 19–62% women [25]. The MRC CR07 trial indicated increased sexual dysfunction for male patients after surgery in general; the additional radiotherapy added only a few points in the sexual dysfunction score [23]. The Dutch TME trial showed an overall sexual dysfunction rate of ∼76% in men, with a mean increase of 8 in the sexual dysfunction score in the radiotherapy group [26]. Worsened sexual functions were reported by 62% of the women after therapy in this trial, with a mean increase of 10 in the score in the radiotherapy group. In the Polish trial, patients completed a questionnaire regarding changes in their sexual life after SCPRT and resection. In this group, 80% of the men and 41% of the women reported a reduction of sexual activity after treatment [13]. Hendren and colleagues reported 61% of women and 91% of men were sexually active before treatment and 32% and 50% postoperatively [27].

The reported toxicity in the presented patient cohort in our study (44% in men and 20% in women) is situated at the lower edge of the incidence rates in literature. ED rates in literature following rectal cancer therapy range from 29–86% [28–34]. The presented rates in our cohort of 44% sexual dysfunction and 36% ED are within the median range of the available numbers in other trials. Stephens *et al*. analyzed the quality of life in the MRC-CR07 trial and concluded that sexual dysfunction is the major chronic side effect among male patients, with resection as the dominant cause [23]. Within the Dutch TME trial, 990 patients were prospectively evaluated regarding risk factors for and incidence of sexual dysfunction. Lange and colleagues identified 79.8% of the men in the radiotherapy arm with dysfunctions—almost twice as many as in our cohort [31]. In contrast to this, similar low rates of ejaculation problems were reported by Breukink *et al*. at 11%, compared with 7.4% here [30].

Some studies used the IIEF (international index of erectile function), others used categories of mild, moderate and complete ED [30, 32–35]. Song *et al*. described a moderate ED rate of 39.7% and a complete rate of 9% after SCPRT and TME. When these numbers are evaluated, the high prevalence of ED among patients above the age of 50 has to be considered. Screening tests among almost 1000 men without pelvic surgery or radiotherapy found a 58.1% ED rate among 60–69 year old men and 79.4% among men 70 years or older [36]. In our results, about a third of the patients (37%) experienced a negative influence on their sexual function by combined modality treatment.

Our reported rate of sexual dysfunction among women younger than 70 years of 35% is comparable with that of other published studies [25]. Overall, reported sexual dysfunctions among women appear to be less frequent compared with men [27, 31]. In our cohort, three patients reported early amenorrhea. Tekkis *et al*. prospectively analyzed sexual function among 295 women after multimodal rectal cancer therapy [37]. They found a rate of dyspareunia of 27.3% five years after abdominal resection and 62% after abdominoperineal resection. In the multivariate analysis, pelvic radiotherapy was found to be a significant risk factor. In the Dutch TME trial, 59.1% of preoperatively sexually active women stated a new or worsened dyspareunia [31]. The dyspareunia rate in our patient group of 12% is clearly lower than that, but this number is for all women and did not exclude patients without sexual activity prior to therapy.

The real rate of therapy-induced problems among sexually inactive women can only be estimated. This may contribute to the fact that the dysfunction rates appear to be lower in the female population compared with the male population.

It is important to note that side effects on sexual life have to be considered in an elderly population as well. Epidemiological studies have indicated that 26% of those questioned from the 75–84 years age interval would have been sexually active [38]. For the age group 65–74 years, which represents the median age interval of our patient group, 53% of people are indicated as having an active sexual life. Schmidt *et al*. found significantly more impact on quality of life and sexual problems for women younger than 70 years [39]. Nevertheless, higher age groups also report impaired sexual functions [40].

A stoma was strongly correlated with absence of sexual activity (57% of people without a stoma were sexually active, whereas only 16% of people with a stoma were active). Similar results have been obtained by others [25, 37, 39]. This is understood as being caused by both operation trauma and psychological effects.

Our reported rate of hip or pelvic fractures of 2% is comparable with that of other trials, in which fracture rates were between 0.7 and 5% [7, 12, 22].

## CONCLUSION

SCPRT combined with total mesorectal excision resulted in excellent LC rates and produced a low rate of Grade 3 chronic side effects. SCPRT can improve OS for Stage III patients. Stool incontinence and sexual dysfunction occur in a considerable percentage of patients (caused by the combined treatments of radiotherapy and surgery) and thus affect their quality of life.

## CONFLICT OF INTEREST

The authors declare that there are no conflicts of interest.

## FUNDING

Funding to pay the Open Access publication charges for this article was provided by the University of Heidelberg decreased side effects.
